# In Critically Ill Patients, Serum Procalcitonin Is More Useful in Differentiating between Sepsis and SIRS than CRP, Il-6, or LBP

**DOI:** 10.1155/2011/594645

**Published:** 2011-05-15

**Authors:** Iwan A. Meynaar, Wouter Droog, Manou Batstra, Rolf Vreede, Paul Herbrink

**Affiliations:** ^1^Intensive Care Unit of the Reinier de Graaf Groep, Reinier de Graafweg 3-11, 2625AD Delft, The Netherlands; ^2^Department of Medical Immunology of the Reinier de Graaf Groep, Reinier de Graafweg 3-11, 2625AD Delft, The Netherlands; ^3^Department of Microbiology of the Reinier de Graaf Groep, Reinier de Graafweg 3-11, 2625AD Delft, The Netherlands

## Abstract

We studied the usefulness of serum procalcitonin (PCT), interleukin-6 (IL-6), lipopolysaccharide binding protein (LBP) levels and C-reactive protein (CRP) levels, in differentiating between systemic inflammatory response syndrome (SIRS) and sepsis in critically ill patients. *Methods*. In this single centre prospective observational study we included all consecutive patients admitted with SIRS or sepsis to the ICU. Blood samples for measuring CRP, PCT, IL-6 and LBP were taken every day until ICU discharge. *Results*. A total of 76 patients were included, 32 with sepsis and 44 with SIRS. Patients with sepsis were sicker on admission and had a higher mortality. CRP, PCT, IL-6 and LBP levels were significantly higher in patients with sepsis as compared to SIRS. With PCT levels in the first 24 hours after ICU admission <2 ng/mL, sepsis was virtually excluded (negative predictive value 97%). With PCT >10 ng/mL, sepsis with bacterial infection was very likely (positive predictive value 88%). PCT was best at discriminating between SIRS and sepsis with the highest area under the ROC curve (0.95, 95% CI 0.90–0.99). *Discussion*. This study showed that PCT is more useful than LBP, CRP and IL-6 in differentiating sepsis from SIRS.

## 1. Introduction

Critically, ill patients often present with the systemic inflammation syndrome (SIRS), and these patients are treated with supportive therapy [1]. SIRS is commonly seen after major surgery, after trauma, with severe inflammation as with pancreatitis and with infection. If SIRS is due to infection, the diagnosis is sepsis and supportive therapy alone is insufficient. The diagnosis of sepsis warrants specific and rapid therapy including early administration of antibiotics and control of the source of the sepsis [2]. In addition, sepsis has a worse prognosis than SIRS. Differentiating between sepsis and SIRS is of utmost importance, and this is a common dilemma for the intensivist. Many biomarkers have been proposed and tested in a clinical setting, but this search has not provided a test that is both widely accepted and enables the bedside clinician to confidently confirm or reject the diagnosis of sepsis [3]. Serum levels of procalcitonin (PCT) are elevated in patients with sepsis, and the usefulness of PCT in diagnosing sepsis has been studied extensively with conflicting results [4–7]. The biological function of PCT is not known. Normal serum levels are below 0.5 ng/mL, and patients with levels above 2 ng/mL are supposedly at risk for sepsis [8]. Serum half-time is 24–36 hours. In their meta-analysis of 33 studies including almost 4000 patients, Uzzan et al. conclude that PCT is superior to CRP in differentiating between sepsis and SIRS and these authors favour the routine use of PCT to help differentiate between SIRS, and sepsis [7]. On the contrary, Tang et al. review 18 studies including 2097 patients to conclude that PCT cannot reliably differentiate sepsis from other causes of SIRS and they argue against the routine use of PCT to aid in differentiating sepsis from SIRS [6]. Interleukin-6 (IL-6) is an important mediator of the acute phase reaction in response to inflammation in sepsis. IL-6 is produced in response to TNF-alpha stimulation. In the liver, IL-6 induces synthesis of acute phase proteins like CRP. The normal range of IL-6 serum concentration is <5.9 pg/mL. Following inflammation serum levels of IL-6 have been shown to rise within one hour, before CRP levels do and even before the onset of fever. IL-6 values above 500 pg/mL were found in patients with sepsis [8]. Lipopolysaccharide binding protein (LBP) is an acute-phase protein produced by the liver in response to circulating bacterial endotoxins or lipopolysaccharide (LPS). LPS is a constituent of the outer coat of Gram-negative bacteria. LBP facilitates the binding of LPS to the LPS receptor on monocytes, resulting in monocyte activation and cytokine production (e.g., TNF alpha and IL-6). Elevated levels of serum LBP have been reported in Gram-negative, Gram-positive, and fungal infections, but not in viral infections [9]. The normal range of LBP serum concentration is 5 to 10 *μ*g/mL [8]. In patients with sepsis LBP levels rise to over 50 *μ*g/mL in about 36 hours [8, 10]. CRP is produced by the liver in response to stimulation by several cytokines one of which is IL-6 [8]. CRP is measured routinely in hospitalized patients although it is generally recognized not to be specific for sepsis or infection as it is elevated in infectious and noninfectious states [8, 11]. CRP in itself has pro- and anti-inflammatory properties. 

The objective of this prospective single centre cohort study was to see if PCT, IL-6, and LBP are useful in differentiating between sepsis and SIRS in critically ill patients.

## 2. Methods

### 2.1. Study Design and Participants

This single-centre prospective observational study was performed from February 1 through April 30, 2009, in the 10-bed mixed ICU of the Reinier de Graaf Hospital. The Reinier de Graaf Hospital is a 500-bed nonacademic teaching hospital. All specialties except neurosurgery and cardiac surgery are available. The hospital has an ICU-based medical emergency team. All consecutive patients admitted to the ICU were included if they were expected to be treated in the ICU for more than 24 hours. If a patient had neither SIRS nor sepsis, this patient was excluded from the study. If a patient had more than one ICU episode in the study period, only the first episode was included in the study.

### 2.2. Sepsis and SIRS

According to the standard definition patients with at least two of the following four criteria: (1) fever (>38°C)or hypothermia (<36°C), (2) tachypnoea (>20/min), (3) tachycardia (>90/min), or (4) leucopenia (<4.0×10^9^/L), leucocytosis (>12.0×10^9^/L or a leftward shift (>10% immature granulocytes) were defined as having SIRS [1]. If SIRS was accompanied with bacterial infection as proven by cultures or on clinical grounds a patient was defined as having sepsis [1]. During treatment the doctors in charge were not blinded but did not use PCT, IL-6, or LBP results for clinical decision making. The final diagnosis of sepsis or SIRS was made at a later stage from the patients' records and blinded to PCT, LBP, and IL-6 results. Due to the small numbers we did not differentiate between sepsis, severe sepsis, or septic shock.

### 2.3. Data Collection

On admission to the ICU patient characteristics and illness severity scores were documented. Blood samples for measuring CRP, PCT, IL-6, and LBP were taken on admission and subsequently at 6 am every morning until ICU discharge.

### 2.4. Laboratory Measurements

PCT levels were measured using a time resolved amplified cryptate emission (TRACE) assay (Kryptor Compact; Brahms, Germany). Intra- and interassay coefficients of variation as determined in our laboratory were, depending on the sample concentration, between 2 and 5%. The best cut-off value of PCT in discriminating between septic and nonseptic patients is still unclear, but most authors suggest a cut-off value of around 2 ng/mL. Additionally, we arbitrarily chose a higher cut-off value of 10 ng/mL to see if we could increase the positive predictive value of PCT, that is, to find a cut-off value above which sepsis could be confirmed without doubt. LBP and IL-6 levels were measured using a solid phase, enzyme-labelled chemiluminescent immunometric assay (IMMULITE 2000; Siemens healthcare, The Netherlands). Interassay coefficients of variation as determined in our laboratory were, depending on the sample concentration, between 3.9 and 14.3% for IL-6 and between 5.9 and 6.2% for LBP. CRP levels were measured using an immunoturbidometric assay (Architect C16000, Abbott Laboratories). Interassay coefficients of variation, as determined by the manufacturer, ranged from 0.44% to 1.25%. A cut-off level of 50 mg/L is commonly used clinical practice.

### 2.5. Statistical Analysis

For statistical analysis, we used SPSS 18.0 (SPSS Inc., Chicago, IL). Comparisons between SIRS and sepsis patient characteristics were made using the Mann Whitney *U* test for continuous variables and Chi square test for categorical variables. To compare the usefulness of different markers in diagnosing sepsis, we used the highest value of each marker in the first 24 hours of admission for each patient. Sensitivity, specificity, positive and negative predictive values, and diagnostic odds ratios (DOR) were calculated, and a receiver-operating characteristic (ROC) curve was made. The DOR can be used to represent test performance in one single figure. The DOR is defined as [sensitivity/(1 − sensitivity)]/[(1 − specificity)/specificity] and can be read as the ratio of the odds of disease with a positive test relative to the odds of disease with a negative test. DOR can range from 0 to infinity, with a higher value indicating a better performance of the test. A DOR of 1 means that the test is useless, a DOR > 25 represents a useful test and a DOR > 100 represent a good test [6, 12, 13]. 

## 3. Results

A total of 76 patients were included in the study; 32 with sepsis and 44 with SIRS. Patient characteristics are summarized in Table 1. As expected, patients with sepsis had significantly higher illness severity scores on admission as compared to patients with SIRS. Patients with sepsis were more often ventilated and put on renal replacement therapy. Both mortality and length of stay in the ICU were higher in patients with sepsis.

**Figure 1:**
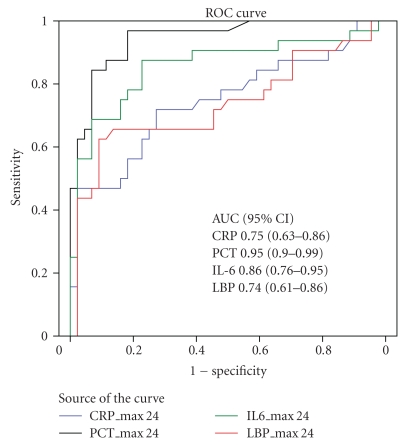
ROC curve and areas under the curve for diagnosing sepsis with the highest values in the first 24 hours after ICU admission for CRP, PCT, IL-6, and LBP.

To compare the usefulness of CRP, PCT, IL-6, and LBP in differentiating sepsis from SIRS we used the highest level of each in the first 24 hours of admission. On admission CRP, PCT, IL-6, and LBP levels were all significantly higher in patients with sepsis in comparison to patients with SIRS (Table 2). The differences were not equal: median CRP and LBP levels in sepsis were about 1.5 to 2 times higher in sepsis, whereas median PCT and IL-6 were about 10 times higher in sepsis as compared to SIRS. Interquartile ranges for IL-6 and PCT did not overlap, but they did overlap for CRP and LBP. With cut-off values of 2 and 10 ng/mL for PCT, 50 *μ*g/mL for CRP, 50 pg/mL for IL-6, and 30 *μ*g/mL for LBP sensitivity, specificity, and predictive values are presented in Tables 3 and 4. We found that PCT below 2 ng/mL makes sepsis highly unlikely (negative predictive value of 97%) and PCT above 10 ng/mL makes sepsis very likely (positive predictive value 88%). PCT has a diagnostic odds ratio of 120.6 with cut-off value of 2 ng/mL.  Figure 1 shows ROC curves and areas under the curve for CRP, PCT, IL-6, and LBP for diagnosis of sepsis as opposed to SIRS. The area under the ROC curve is significantly higher for PCT as compared to IL-6, LBP, and CRP.

**Figure 2:**
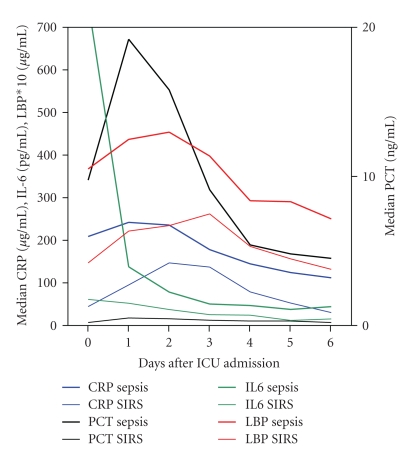
Median CRP, PCT, LBP, and IL-6 levels in patients with sepsis and SIRS during ICU admission.

In patients with sepsis, maximum values for IL-6 were reached on day 0, for PCT and CRP on day 1 and for LBP on day 2 (Figure 2). Il-6 levels in patients with sepsis decline rapidly after day 1. The difference in PCT levels between sepsis and SIRS patients is maintained at least until day 3 or 4.

## 4. Discussion

This single-centre prospective observational study showed that serum PCT levels are more valuable than serum CRP, LBP, and IL-6 levels in discriminating sepsis from SIRS in critically ill patients. PCT has the highest area under the ROC curve and the highest diagnostic odds ratios. If PCT levels in the first 24 hours after ICU admission are below 2 ng/mL, sepsis with bacterial infection is virtually excluded (negative predictive value 97%). If PCT levels in the first 24 hours after ICU admission are above10 ng/mL, sepsis with bacterial infection is very likely (positive predictive value 88%). Comparing positive and negative predictive values, PCT seems to be even more useful in excluding sepsis than in diagnosing sepsis. LBP, CRP, and IL-6 had lower positive and negative predictive values. IL-6 is the second best after PCT but the sharp decline of IL-6 levels after admission in patients with sepsis suggests that late sampling may easily cause false negative results. 

A particular strength of this study it that is compares not only PCT but also LBP and Il-6 with standard CRP. The fact that this is a pragmatic real-life study adds to its strength, but we recognize that this also induces serious limitations. The study is relatively small, one of the smaller studies on this subject, and was performed in a single centre where no cardiac surgery or neurosurgery patients are treated. Also we found a rather high DOR for PCT (120.6 and 26.1 for cut-off values of 2 and 10 ng/mL, resp.) as compared to the pooled DOR of 7.79 found by Tang et al. It is quite possible that due to the limitations of our study we have overestimated the diagnostic properties of PCT. Although PCT, IL-6, and LBP values were not used for treatment decisions, treating physicians were not blinded to the test results and this may have induced bias. The eventual diagnosis of sepsis versus SIRS was made by the physicians who were previously involved in treatment of the patients and this may have resulted in hindsight bias or information bias. Since patients with severe sepsis are more likely to be admitted to the ICU than patients with mild SIRS we may have missed patients with mild SIRS but with elevated PCT that went unnoticed, resulting in selection bias. 

The real-life value of an additional diagnostic tool is not easily estimated. The process of reaching a diagnosis is not entirely rational. In everyday practice clinicians use many clues (signs, symptoms, epidemiology, experience, etc.) to reach a (differential) diagnosis. The value of a test depends on how probable the clinician thinks a certain diagnosis is beforehand (pretest probability). If the clinician is not in doubt any additional test is useless. If the doctor is in doubt and the additional test brings more certainty on rejecting or accepting a diagnosis (posttest probability), the test is useful in this case. In addition, sometimes unlikely options are taken into account because of the grave consequences of neglecting such an option. The influence of an additional test on diagnosis and subsequent action depends not only on the pretest and posttest probability, but also on our willingness to act in accordance with the additional test result. In the context of this paper, this would for instance mean: would we be willing to withhold a patient admitted for suspected sepsis antibiotics, if his PCT turned out to be low? The answer depends on our estimation of the likeliness of sepsis (pretest probability) but also on our willingness to accept the risk of not giving antibiotics. The point that we want to make is that the answer is not purely mathematical and cannot be given by just studying diagnostic properties of the test. We would therefore urge clinicians to see for themselves if PCT could be of value in differentiating SIRS from sepsis in their practice, as we have done. Incorporating PCT into clinical practice could improve decision making especially in patients with conflicting clues on the presence or absence of sepsis. PCT could also be valuable as a means to reduce the length of antibiotic treatment but that is outside the scope of this paper [14]. 

## 5. Conclusion

This study showed that PCT levels are of value in differentiating between sepsis and SIRS in critically ill patients and more helpful than CRP, IL-6, or LBP levels. Especially during the first 24 hour of admission, PCT levels can help determine the course of action to improve outcome, reduce mortality, and prevent unnecessary diagnostic and therapeutic measures.

Although PCT is the best biomarker to distinguish sepsis from SIRS its diagnostic properties do not justify clinical decision making based on PCT alone. The diagnosis of sepsis still requires integration of multiple clinical data.

##  Conflict of Interests

None of the authors has any conflict of interests to declare.

## Figures and Tables

**Table 1:** Patient characteristics.

	All	Sepsis	SIRS	P**
Number	76	32	44	—
Age*	66 (56–78)	68 (56–78)	65 (54–75)	ns
APACHE IV score*	57 (44–78)	70 (51–106)	53 (41–63)	<.001
APACHE IV exp mort (median)	18%	25%	16%	<.001
Medical	35	17	18	—
Planned surgery	25	4	21	.002
Emergency surgery	16	11	5	—
Ventilated	51	24	27	ns
days on ventilator*	4 (2–8)	6.5 (3.2–11)	2 (2–6)	.018
Renal replacement therapy	10	9	1	.001
Source of sepsis:				
gastrointestinal	—	17	—	—
pulmonary	—	8	—	—
other	—	7	—	—
ICU LOS***	3.3 (1.7–7.0)	6.4 (2.4–10.8)	2.7 (1.5–5.8)	.024
Hospital LOS***	14 (7–30)	23 (8–36)	12 (7–21)	.082
ICU mortality	7 (9%)	5 (16%)	2 (5%)	.099
Hospital mortality	14 (18%)	9 (28%)	5 (11%)	.063

*Median, interquartile range.

**For difference between sepsis and SIRS, Mann Whitney *U* test for continuous variables and Chi square test for categorical variables.

***LOS: length of stay.

**Table 2:** The highest levels of CRP, PCT, IL-6, and LBP in the first 24 hours of ICU treatment.

	All	Sepsis	SIRS	P**
CRP* (*μ*g/mL)	117 (56–194)	179 (88–297)	80 (52–152)	<.001
PCT* (ng/mL)	2.2 (0.3–20.3)	24.3 (6.6–57.2)	0.5 (0.2–1.1)	<.001
IL-6* (pg/mL)	153 (41–750)	1463 (243–12951)	54 (25–149)	<.001
LBP* (*μ*g/mL)	19.1 (12.6–31.7)	30.9 (14.7–41.5)	16.3 (10.8–22.2)	.001

*Median, interquartile range.

**Mann Whitney *U* test for difference between sepsis and SIRS.

**Table 3:** Test results using the highest value of the biomarkers within the first 24 hours of admission on the ICU.

		Sepsis	No sepsis	All
CRP (cut-off value 50 *μ*g/mL)	Test +	28	34	62
	Test −	4	10	14
PCT (cut-off value 2 ng/mL)	Test +	31	9	40
	Test −	1	35	36
PCT (cut-off value 10 ng/mL)	Test +	21	3	24
	Test −	11	41	52
IL-6 (cut-off value 50 pg/mL)	Test +	29	26	55
	Test −	3	18	21
LBP (cut-off value 30 *μ*g/mL)	Test +	17	4	21
	Test −	15	40	55

All patients		32	44	76

Test +: number of patients with marker-level equal or above cut-off value.

Test −: number of patients with marker-level below cut-off value.

**Table 4:** Sensitivity, specificity, predictive values, and diagnostic odds ratios using the highest values of all biomarkers within the first 24 hrs of ICU admission.

	Sensitivity	Specificity	PPV	NPV	DOR (95% CI)
CRP cut-off value 50 *μ*g/mL	88%	23%	45%	71%	2.1 (0.6–7.3)
PCT cut-off value 2 ng/mL	97%	80%	78%	97%	120.6 (14.4–1006)
PCT cut-off value 10 ng/mL	66%	93%	88%	79%	26.1 (6.6–103.8)
IL-6 cut-off value 50 pg/mL	91%	41%	53%	86%	6.7 (1.8–25.4)
LBP cut-off value 30 *μ*g/mL	53%	91%	81%	73%	11.3 (3.3–39.3)

Sensitivity: percentage of septic patients with positive test.

Specificity: percentage of nonseptic patients with negative test.

PPV (positive predictive value): percentage of test-positive patients with sepsis.

NPV (negative predictive value): percentage of test-negative patients without sepsis.

DOR (diagnostic odds ratio): [sensitivity/(1 − sensitivity)]/[(1 − specificity)/specificity]: the ratio of the odds of disease with a positive test relative to the odds of disease with a negative test.
